# Combined Rosiglitazone and Forskolin Have Neuroprotective Effects in SD Rats after Spinal Cord Injury

**DOI:** 10.1155/2018/3897478

**Published:** 2018-06-21

**Authors:** Qing-qi Meng, Wei Lei, Hao Chen, Zhen-cheng Feng, Li-qiong Hu, Xing-liang Zhang, Siming Li

**Affiliations:** ^1^Department of Orthopedics, Guangzhou Red Cross Hospital, Jinan University, 396 Tongfu Road, Guangzhou 510120, China; ^2^Laboratory Research Center, Guangdong Medical University, Zhanjiang 524001, China; ^3^Department of Gastroenterology, Guangdong General Hospital and Guangdong Academy of Medical Sciences, Guangzhou 51000, China

## Abstract

The peroxisome proliferator-activated receptor gamma (PPAR-*γ*) agonist rosiglitazone inhibits NF-*κ*B expression and endogenous neural stem cell differentiation into neurons and reduces the inflammatory cascade after spinal cord injury (SCI). The aim of this study was to explore the mechanisms underlying rosiglitazone-mediated neuroprotective effects and regulation of the balance between the inflammatory cascade and generation of endogenous spinal cord neurons by using a spinal cord-derived neural stem cell culture system as well as SD rat SCI model. Activation of PPAR-*γ* could promote neural stem cell proliferation and inhibit PKA expression and neuronal formation* in vitro.* In the SD rat SCI model, the rosiglitazone + forskolin group showed better locomotor recovery compared to the rosiglitazone and forskolin groups. MAP2 expression was higher in the rosiglitazone + forskolin group than in the rosiglitazone group, NF-*κ*B expression was lower in the rosiglitazone + forskolin group than in the forskolin group, and NeuN expression was higher in the rosiglitazone + forskolin group than in the forskolin group. PPAR-*γ* activation likely inhibits NF-*κ*B, thereby reducing the inflammatory cascade, and PKA activation likely promotes neuronal cell regeneration.

## 1. Introduction

Acute spinal cord injuries (SCI) are severe and often permanent and can prove expensive for patients and the community. SCI is the highest cause of death in young people (15–29 years of age) [1]. There are currently no treatments which have a proven positive effect on neurological outcome [2, 3], following acute SCI. Spinal cord injury results in primary initial damage and subsequent secondary damage [4]. The secondary damage due to early inflammatory responses after spinal cord injury is the main cause for neurological defects and exacerbates the initial degree of injury [5] and the secondary damage usually caused by ischemia, cellular and tissue edema, and oxidative damage [6].

In our previous research, we found that activation of peroxisome proliferator-activated receptor-gamma (PPAR-*γ*) could promote locomotor recovery after spinal cord injury [7]. PPAR-*γ* is a PPAR nuclear receptor subtype. There are two types of PPAR-*γ* ligands: natural ligands such as fatty acids and eicosanoid derivatives [8] and synthetic ligands such as thiazolidinediones ketones (thiazolidinedione, TZD), of which rosiglitazone (rosiglitazone) and pioglitazone (pioglitazone) are used in clinical medicine as a treatment for type 2 diabetes [9]. Rosiglitazone is part of the thiazolidinediones nuclear hormone receptor superfamily and is known for its anti-inflammatory actions via the activation of PPAR-*γ*. Rosiglitazone attenuates the expression of proinflammatory genes and cytokine production by regulating ligand activation of transcription factors [10, 11]. And rosiglitazone has neuroprotective effects in neurodegenerative diseases and spinal cord injury [12, 13]. We previously reported that application of the PPAR-*γ* agonist rosiglitazone could significantly inhibit activation of the key inflammatory cascade target nuclear transcription factor kappa B (nuclear factor kappa B; NF-*κ*B) after spinal cord injury [7].

NF-*κ*B is extracted from B lymphocytes and is named because of its binding to the immunoglobulin chain gene enhancer sequence-specific *κ*B [14]. The NF-*κ*B family of transcription factors has an essential role in inflammation and innate immunity. The active form of NF-*κ*B is a dimer, typically a P50 / P65 heterodimer, and is the main component of the NF-*κ*B family involved in gene transcription [15]. Members of the NF-*κ*B family participate in many physiological processes, including regulation of the immune response, stimulation of immune cell maturation, and the production and survival of essential transcription factors for B cells [15].

In our preliminary research, we found that PPAR-*γ* could inhibit NF-*κ*B activation and promote proliferation and inhibit neural differentiation of endogenous neural stem cells [7]. The NF-*κ*B and PPAR-*γ* signaling pathways are closely related; PPAR-*γ* activation along with inhibition of the NF-*κ*B-cytokine cascade could protect against dextran sulfate sodium-induced colitis in mice [16]. PPAR-*γ* could also attenuate inflammation via NF-*κ*B inhibition in lipopolysaccharide-induced peritonitis [17]. In addition to functions in glucose and lipid metabolism and cell proliferation and differentiation, PPAR-*γ* can also alleviate the acute and chronic inflammatory response after nerve injury and ameliorate neuronal apoptosis and ischemic brain injury by suppressing NF-*κ*B-driven p22phox transcription [18].

Imielski et al. [19] reported distinct loss of hippocampal dentate gyrus neuronal tissue in NF-*κ*B gene-deficient mice; reactivation of NF-*κ*B could promote the formation of dentate gyrus neurons and neural network and improve neuronal organization and animal behavior. The underlying mechanism is likely related to the activation of cAMP/PKA after activation of NF-*κ*B. cAMP (Cyclic Adenosine Monophosphate) was discovered in 1958 by Earl [20]; increased levels of cAMP lead to a transient enhancement of transmitter release [21] and regulation of cAMP and cGMP together ensures coordinated development of one axon and multiple dendrites. PKA could enhance transmitter release at the synaptic connection between the sensory and motor neuron [22]. PKA is implicated in the growth of axons and survival of newborn neurons [19, 23]. Forskolin is a unique diterpene activator of cyclic AMP-generating systems [24]. Forskolin-mediated activation of adenylate cyclase stimulates nerve regeneration* in vivo* [25]. Sulaiman et al. [26] found that treatment with forskolin alone and in combination with TGF-*β* resulted in an increase in the number of regenerated axons compared with saline-only control.

These findings were consistent with our preliminary findings that activation of PPAR-*γ* could inhibit the expression of NF-*κ*B in a SD rat spinal cord injury model, while inhibiting neuronal survival. We therefore hypothesized that PPAR-*γ* activation in SD rats inhibits neurons via PKA, which is downstream of NF-*κ*B. In* in vitro* experiments, we intend to verify that PPAR-*γ* activation inhibits NF-*κ*B expression, which in turn could inhibit the cAMP/PKA pathway. After confirming the inhibition of cAMP/PKA by PPAR-*γ*, animal experiments will be initiated to determine whether simultaneous activation of PPAR-*γ* and PKA could inhibit inflammation and promote neuronal formation. In this study, we explored the role of PPAR-*γ* and PKA in functional recovery after spinal cord injury in SD rats. We aimed to determine the mechanisms underlying this recovery, as well as the balance between the inflammatory cascade and generation of endogenous spinal cord neurons.

## 2. Materials and Methods

### 2.1. *In Vitro* Experiments

#### 2.1.1. Extraction and Culture of Spinal Cord-Derived Neural Stem Cells

One-day-old SD rats were killed by cervical dislocation and immersed in 75% alcohol. Three sets of eye scissors and tweezers were prepared in sequence and placed in beakers with 75% alcohol, numbered 1, 2, and 3, respectively. The skin was cut using the first scissors, and both sides of the spine were cut from the rib fossa, from the bottom up, to remove the modified fascia and fat tissue attached to the spine by using the second scissors. With 1 mL of serum-free DMEM/F12 medium drawn with a 1 mL syringe, syringe needle was flushed from one end of the spine and washed 3 times repeatedly to flush out the spinal cord. The spinal cord was then cut into 2–3 pieces by using the third pair of scissors, and the pieces of spinal cord tissue were removed into a Petri dish filled with culture medium and subjected to repeated aspiration using a syringe 5 to form a uniform suspension. The spinal cord pieces were sieved through 200-mesh sieve in order to separate them into single cells. After suspension in serum-free DMEM/F12, samples were centrifuged for 5 min (1000 rpm). The cells were stained with trypan blue, counted, and seeded at a density of 1 × 10^6^ cells /ml in culture flasks and incubated at 37 °C in a 5% CO_2_ humidified atmosphere. Culture medium was changed after 3 days and a half. Peculiar “nerve ball” formation was observed using an inverted microscope.

After 5 to 7 days, the spinal cord-derived stem cells were proliferated and expanded* in vitro *for 1 passage. The cells were separated mechanically by using pipettes and 200-mesh sieve and centrifuged for 5 min (1000r/min). The supernatant was discarded, and 2×10^5^/ml spinal cord-derived stem cells were inoculated onto the surface of a sterile-10 Petri dish and incubated in medium containing DMEM/F12 + 2% B27 + 20 ng/ml b FGF + 20 ng/ml EGF. This free-serum culture system was used in order to screen NSCs. Each time medium is changed and every passage flasks were changed to discard the adherent cells. Thus NSCs are naturally purified during the cultivation process. The purity and quality of NSC were calculated by staining with Nestin/DAPI.

Neural stem cells derived from the spinal cord were divided into three groups, namely, the rosiglitazone group, treated with rosiglitazone (1 *μ*mol/L in DMSO [Abcam, ab142179]), the G3335 group, treated with G3335 (PPAR-*γ*-antagonist 0.1 *μ*mol/L) and DMSO, and the vehicle group, treated with DMSO. After 3 days continuous culture, proliferation of NSCs was evaluated by cell number using Cellometer Auto 1000 (USA, Nexcelom).

Neural stem cells were grown in medium supplemented with 10% fetal bovine serum (the medium was used to induce differentiation). The early neuronal marker MAP2 was detected by QRT-PCR and western blot.

#### 2.1.2. QRT-PCR Detection of MAP2, PKA, and PPAR-*γ* Expression

Total RNA was isolated using the Trizol®RNA isolation reagent (Invitrogen) and quantitative data was recorded. cDNAs were reverse-transcribed using the RevertAid First Strand cDNA Synthesis Kit (Fermentas). The target gene fragments were amplified using real time-PCR (RT-PCR). Maxima SYBR Green (Thermo Scientific, Waltham, MA, USA) was used for staining. The target gene expression was normalized to glyceraldehyde-3-phosphate dehydrogenase (GAPDH): primers: MAP2 (forward 5′- AACATACCACCAGCCCTTTG -3 ′, reverse 5′ -GCCTTTCCTTCGTCTTTCCT-3′); PKA (forward 5′-AGCCAAAGCCAAGGAAGATT-3′, reverse 5′-AGCATCACTCGCCCAAAG-3′); PPAR-*γ* (forward 5′-CGAGAAGGAGAAGCTGTTGG-3′, reverse 5′- TCAGCGGGAAGGACTTTATG-3′).

#### 2.1.3. Western Blot Detection of MAP2, PKA, and PPAR-*γ* Expression

Total cellular protein was extracted and 25 *μ*g was used for SDS-PAGE. After blocking the membrane for 30 min, rinsing with TBST buffer three times, and incubation with anti-MAP2 (1:1000; Abcam; ab5392), anti-PKA (1:400; Abcam ab211265), and anti-PPAR-*γ* (1:500; Abcam ab45036) for 12 h at 4 °C, the membranes were washed three times in wash buffer (0.2% gelatin, 0.05% Tween 20 in TBS). The membranes were then incubated for 1 h with horseradish peroxidase-labeled secondary antibody, washed again, and exposed to ECL color development reagents. The membranes were developed using the ChemiDoc-It TM TS2 Imaging System (Bio-Rad), and relative optical density was analyzed using the ImageJ2x software (National Institute of Health, Bethesda, MD, USA).

#### 2.1.4. Detection of cAMP by ELISA

The production of cAMP was determined by ELISA after rosiglitazone treatment. The supernatants from the neural stem cell cultures were harvested, and cAMP level was determined using an ELISA kit (GBD, Co.), according to the manufacturer's instructions.

### 2.2. *In Vivo* Experiments

#### 2.2.1. Animals

**Figure 1:**
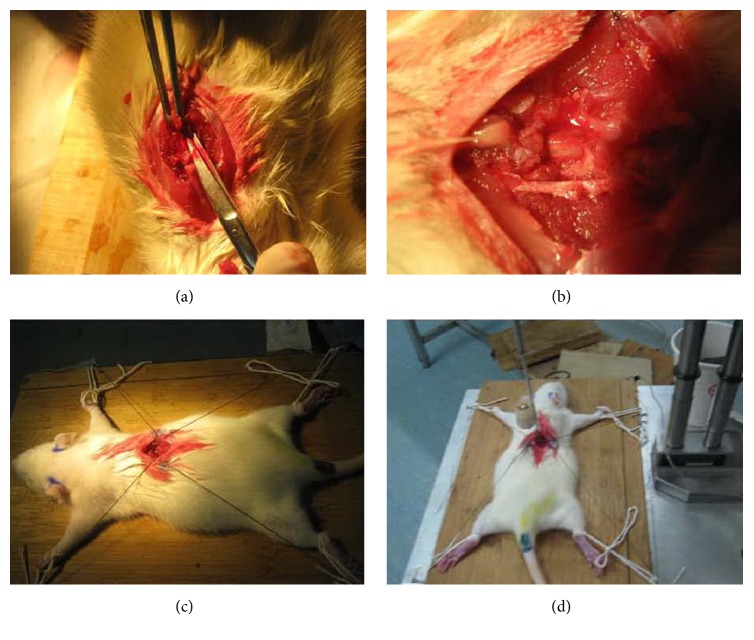
Representative images showing the surgical process in SD rats. (a-b) Dorsal skin preparation and disinfection and exposed T9-T10 spinous lamina with sterile surgical instruments and spinal cord. (c-d) Rats were fixed on an operating table and subjected to instant impact against the spinal apparatus. The animals' bodies shook, with the lower limbs rapidly retracting and bouncing and tail cocking and quickly falling. The impacted local spinal cord surface was dark purple, and hind limbs appeared completely paralyzed postoperatively.

SD rats were housed in a temperature-controlled room at 27°C. Injured rats underwent manual bladder expression twice a day. Under halothane anesthesia [induction, 4%; maintenance, 2% in an oxygen and nitrous oxide (50:50) mixture], SCI was induced in adult female Sprague-Dawley rats by dropping an impactor (10 g weight rod, 2.5 mm in diameter) from a height of 25 mm as previously reported [7], and T9–T10 laminectomy was performed [Figure 1]. SCI rats were randomly divided into 4 groups: vehicle group (n = 3), rosiglitazone group (n = 3), forskolin group (n = 3), and rosiglitazone + forskolin group (n = 3). Rosiglitazone (3 mg/kg, Abcam) or vehicle (0.01 M PBS) was injected i.p., at 5 min, 6 h, and 24 h after SCI. Forskolin (0.1 mg/kg/d; Abcam) was injected i.p. continuously for 7 days.

#### 2.2.2. BBB Score

Motor function recovery after SCI was studied using the Basso–Beattie–Bresnahan locomotor rating scale (BBB scale) [27]. If there was no spontaneous hind limb movement, the score was 0, and a score of 21 indicated normal locomotion. To ensure that all animals began with a score of 21, all rats were tested prior to injury. The rats were then tested at 8 h and on days 1, 3, 7, 14, 21, and 28 after injury (or up to the day the animal was euthanized). Each rat was scored for 4 min by two observers blinded to the study groups.

#### 2.2.3. Spinal Cord Harvest

The three rats from each group were anesthetized by chloral hydrate (10%; 0.33 mL/kg), and the damaged spinal cord (1 cm from the center of the injury point) was immediately removed 28 days after surgery. Spinal cord tissues (T8–T10) were harvested and dissociated in trypsin (0.5 mg/ml) and collagenase (0.5 mg/ml). We separated the debris from cells as described in previous studies [28].

#### 2.2.4. QRT-PCR Analysis of MAP2, NF-*κ*B, and NeuN Expression

Total RNA from 10 mm long spinal cord segments containing the injury epicenter obtained from the 4 groups were extracted using Trizol (Invitrogen, USA) at 28 d after injury. RNA was detected by electrophoresis before reverse transcription. The results showed no degradation; OD260/280 was between 1.8 and 1.9. The cDNA were reverse-transcribed using Revert Aid-First Strand cDNA Synthesis kit (Thermo Scientific). cDNA was used as a template for reverse transcription polymerase chain reaction analysis (RT-PCR) amplification. The 2^-ΔΔCt^ method was used for data analysis. The target gene expression was normalized to that of glyceraldehyde-3-phosphate dehydrogenase (GAPDH): primers: MAP2 (forward 5′- AACATACCACCAGCCCTTTG -3 ′, reverse 5′ -GCCTTTCCTTCGTCTTTCCT-3′); NF-*κ*B (forward 5′AACACTGCCGAGCTCAAGAT-3′, reverse 5′CATCGGCTTGAGAAAAGGAG-3′); NeuN (forward 5′-CGTGGAAAGTGTGGCTGAA-3′, reverse 5′-ACCATAGTCTTCAAAGTCCCG-3′).

#### 2.2.5. Western Blot Analysis of MAP2, NF-*κ*B, and NeuN Expression

Spinal cord tissues (1 cm) were isolated using the lesion site as the epicenter. Spinal cord tissues were homogenized in RIPA lysis buffer with phosphatase and protease inhibitor (benzamidine 1*μ*M; leupeptin 5*μ*g/ml; Na_3_VO_4_ 200*μ*M; sodium pyrophosphate 200*μ*M; and phenylmethylsulfonyl fluoride 200*μ*M). Tissue homogenates were incubated for 20 min at 4°C and centrifuged at 25000g for 30min at 4°C. Proteins were quantified with the bicinchoninic acid protein assay kit (Thermo Scientific) and 10* μ*g of protein was loaded in each lane. Samples were electrophoresed onto a 12% sodium dodecyl sulfate/polyacrylamide gel (SDS/PAGE) and transferred to PVDF membranes (Millipore, Massachusetts, USA). After blocking with 10% BSA in TBST (25 mM Tris-HCl, 0.15 M saline, and 1% Tween 20) at room temperature for 2 h, the membranes were incubated with the following primary antibodies at 4°C overnight: anti-GAPDH (1:1000; Abcam), anti-MAP2 (1:800; Abcam), anti-NF-*κ*B (1:400; Abcam), and anti-NeuN (1:500; Abcam). The membranes were then incubated with horseradish peroxidase-conjugated secondary antibody (1:5000; Abcam) for 2 h at 4°C. During the experiment, the low-temperature environment is strictly guaranteed, and protease inhibitors are added to ensure protein quality.

The membranes were developed using the ChemiDoc-It TM TS2 Imaging System, and relative optical density was analyzed using Quantity one v4.4 software (Bio-Rad).

#### 2.2.6. PKA Determination

The production of PKA was determined by ELISA. The supernatants from spinal cord tissue were harvested, and PKA expression was determined using an ELISA kit (GBD, Co.), according to the manufacturer's instructions.

### 2.3. Statistical Analysis

All statistical analyses were conducted using SPSS 14.0 software. The BBB scores for each animal were averaged and used to determine the group mean each day. The BBB score data were expressed as mean ± SD. Comparisons among groups were performed by one-way analysis of variance (ANOVA) followed by a Tukey–Kramer multiple comparisons post hoc test.

## 3. Results

### 3.1. Neural Stem Cell Properties of NSCs Derived from Rat Spinal Cord

**Figure 2:**
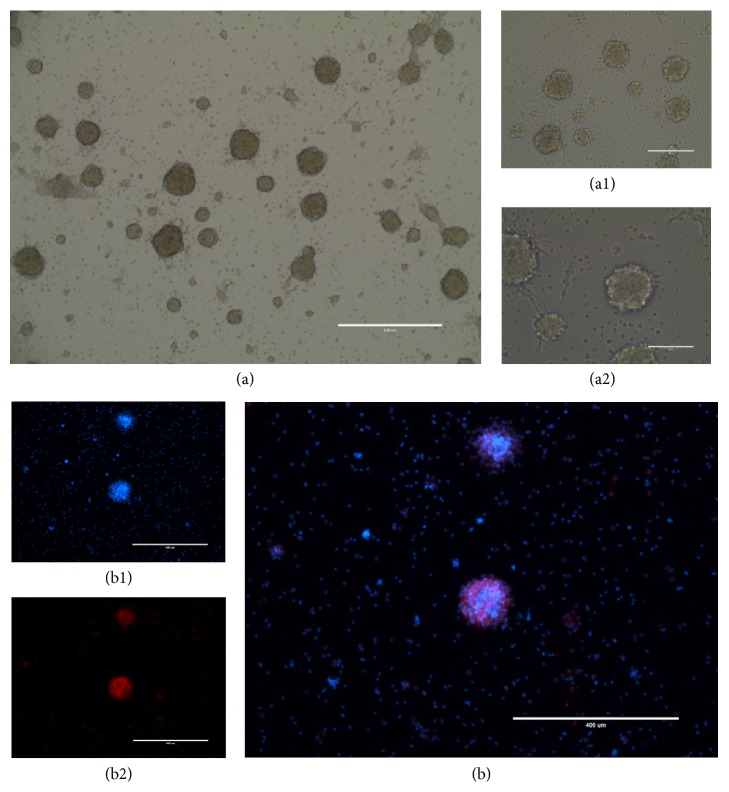
Neural stem cells from rat spinal cord. (a)-(a1)-(a2) Cells gather into neurospheres. (b1) DAPI immunofluorescence staining. (b2) Nestin-CY3 immunofluorescence staining. (b) Nestin-CY3-DAPI-immunofluorescence staining (merge). Immunofluorescence staining was performed to identify neural stem cells, Nestin /DAPI >90%. Each experiment was repeated three times.

Under the light microscope, the primary neural cells appeared round, with small cell bodies and single scattered shiny halos. Most of the cells grew in suspension and formed neural stem cell spheres, while some cells grew adherently. At 3-5 days, dozens of cloned spheres were formed by the suspension of cell clusters, and the cloned spheres showed spherical shape with no obvious protrusions. Three days after inoculation, the NSCs derived from rat spinal cord rapidly proliferated to form cellular spheres or neural spheres (Figure 2(a)-(a1)-(a2)). Immunofluorescence staining showed Nestin expression in the neurospheres, and cytoplasm coloring was seen. Immunofluorescence staining was performed to identify neural stem cells, Nestin /DAPI >90% (Figure 2(b1)-(b2)-(b1)).

### 3.2. *In Vitro *Activation of PPAR-*γ* Could Promote Neural Stem Cell Proliferation

Table 1 shows the number of ENSCs after PPAR-*γ* activation. Second generation of NSCs were selected from each group and cultured after adding rosiglitazone (1 *μ*mol/L) or G3335 (0.1 *μ*mol/L). Cells were cultured for 3 days. NSCs of all three groups proliferated, and the NSC proliferation in the rosiglitazone group was significantly greater than that in the negative control group (*P *< 0.05, n=3). NSC proliferation in the G3335 group was clearly neutralized. There was no significant difference in NSC number between the negative control and rosiglitazone + G3335 groups.

### 3.3. *In Vitro *Activation of PPAR-*γ* Could Inhibit PKA Expression and Neuronal Formation in Spinal Cord-Derived Neural Stem Cells

**Figure 3:**
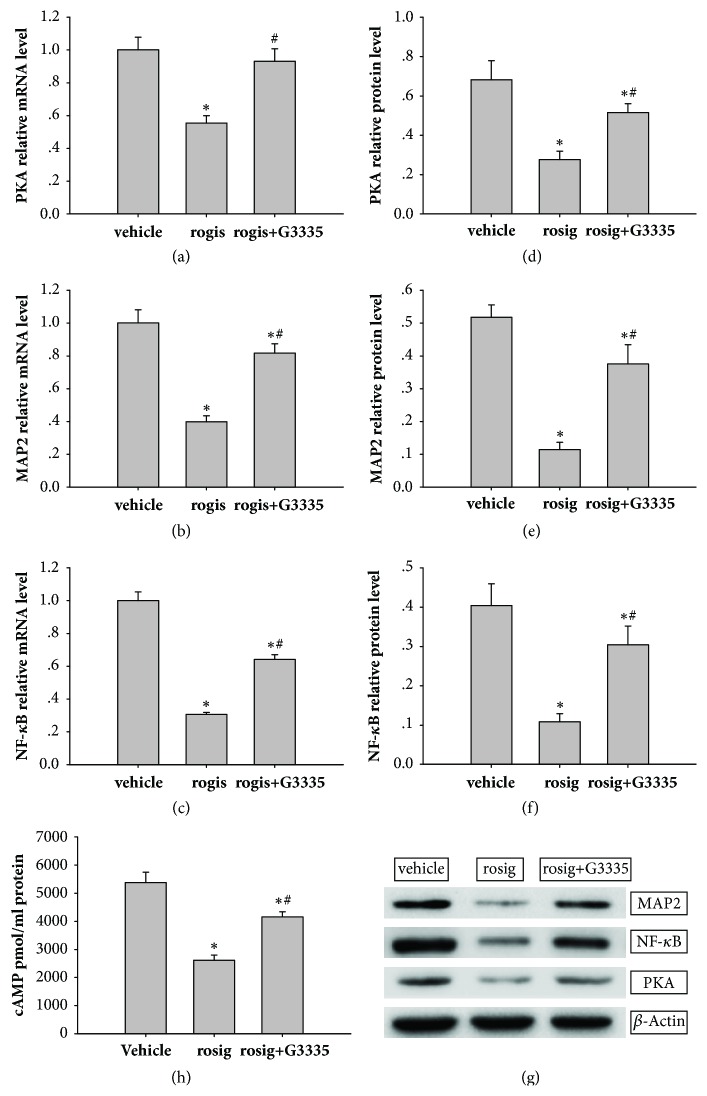
The mRNA and protein expression of PKA, MAP2, and NF-*κ*B. (a) Relative PKA mRNA expression level. (b) MAP2 relative mRNA expression level. (c) Relative NF-*κ*B mRNA expression level. (d) Relative PKA protein expression level. (e) Relative MAP2 protein expression level. (f) Relative NF-*κ*B protein expression level. (g) Representative western blots showing the effects of rosiglitazone and G3335 on MAP2, NF-*κ*B, and PKA. (h) cAMP expression level. Data were expressed as means±SD (n = 3). Each experiment was repeated at least three times: *∗* P < 0.05, compared with vehicle group, and # P < 0.05, compared with rosiglitazone group (one-way ANOVA followed by a Tukey–Kramer multiple comparisons posttest).

We examined the mRNA expression of PKA (Figure 3(a)), MAP2 (Figure 3(b)), and NF-*κ*B (Figure 3(c)) in the three groups. PKA mRNA expression in the rosiglitazone group (0.55 ± 0.04) was lower than that in the vehicle (1.00 ± 0.08), and when the activation of PPAR-*γ* is blocked, the expression of PKA is restored in rosiglitazone + G3335 (0.93 ± 0.08) groups (P < 0.01; n = 3). The expression of MAP2 mRNA in the rosiglitazone group (0.40 ± 0.04) was lower than that in the vehicle (0.82 ± 0.06), and when the activation of PPAR-*γ* is blocked, this inhibition could be partly reversed in rosiglitazone + G3335 (1.00 ± 0.08) groups (P < 0.01; n = 3). Compared with the vehicle (1.00 ± 0.05) and rosiglitazone + G3335 (0.64 ± 0.03) groups, the expression of NF-*κ*B mRNA significantly decreased with the activation of PPAR-*γ* in the rosiglitazone group (0.31 ± 0.002; P < 0.01; n = 3).

In the WB experiment, we observed similar trends of indicators in each group.

The activation of PPAR-*γ* could inhibit PKA expression (Figures 3(d) and 3(j)) expression in the rosiglitazone group (0.28 ± 0.04) which was lower than that in the vehicle (0.68 ± 0.10) and rosiglitazone + G3335 (0.51 ± 0.05) groups (P < 0.01; n = 3).

The expression of neuronal formation marker MAP2 protein (Figures 3(e) and 3(j)) was significantly decreased after adding rosiglitazone (0.11 ± 0.02) and was lower in the rosiglitazone group than in the rosiglitazone + G3335 (0.38 ± 0.06) and vehicle (0.52 ± 0.04) groups (P <0.01; n = 3).

Compared with the vehicle (0.40 ± 0.06) and rosiglitazone + G3335 (0.30 ± 0.05) groups, the expression of NF-*κ*B protein (Figures 3(f) and 3(j)) was significantly decreased in the rosiglitazone group (0.11 ± 0.02; P < 0.01; n = 3, respectively).

In ELISA experiment, cAMP levels in cells treated with rosiglitazone (rosiglitazone group) (2614.77±182.29 ) are lower than vehicle group (5380.88±367.74). After G3335 was used to block the activation of PPAR-*γ*, the cAMP levels in rosiglitazone + G3335 group (4153.45±189.06) were higher than rosiglitazone group (Figure 3(h)).

### 3.4. Simultaneous Activation of PPAR-*γ* and PKA Was Most Effective in Promoting Locomotor Recovery

**Figure 4:**
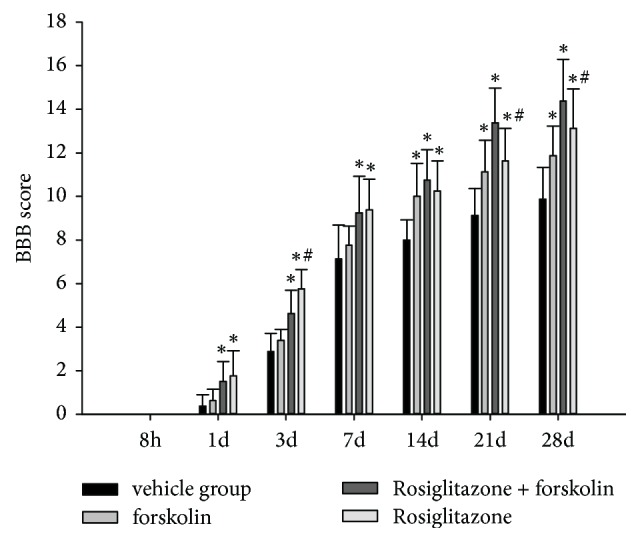
BBB scores after SCI. Data were expressed as means ± SD (n = 3). Each experiment was repeated at least three times. ^*∗*^ P < 0.05, compared with the vehicle group;^ #^ P < 0.05, compared with rosiglitazone + forskolin group.

All rats had a BBB score of 21 before SCI and lost locomotor function 8 h after SCI. At the 24 h point, the BBB scores of all the groups were still not >2. On day 3, compared with the vehicle and rosiglitazone + forskolin groups, the rosiglitazone group showed significantly higher BBB scores (P < 0.05; n = 8). On days 21 to 28, compared with the vehicle group, the rosiglitazone + forskolin group showed significantly higher BBB scores (P < 0.05; n = 8); the rosiglitazone + forskolin group also showed higher BBB scores than those of the forskolin or rosiglitazone groups, respectively (P < 0.05; n = 8). On day 28, all the groups showed the highest BBB scores since SCI (BBB score: 9.88 ± 1.46, 11.88 ± 1.36, 15.38 ± 1.85, and 13.13 ± 1.81, respectively, for the vehicle, forskolin, rosiglitazone + forskolin, and rosiglitazone groups; P < 0.05; n = 8) (Figure 4).

### 3.5. mRNA Expression of MAP2, NeuN, and NF-*κ*B following SCI

**Figure 5:**
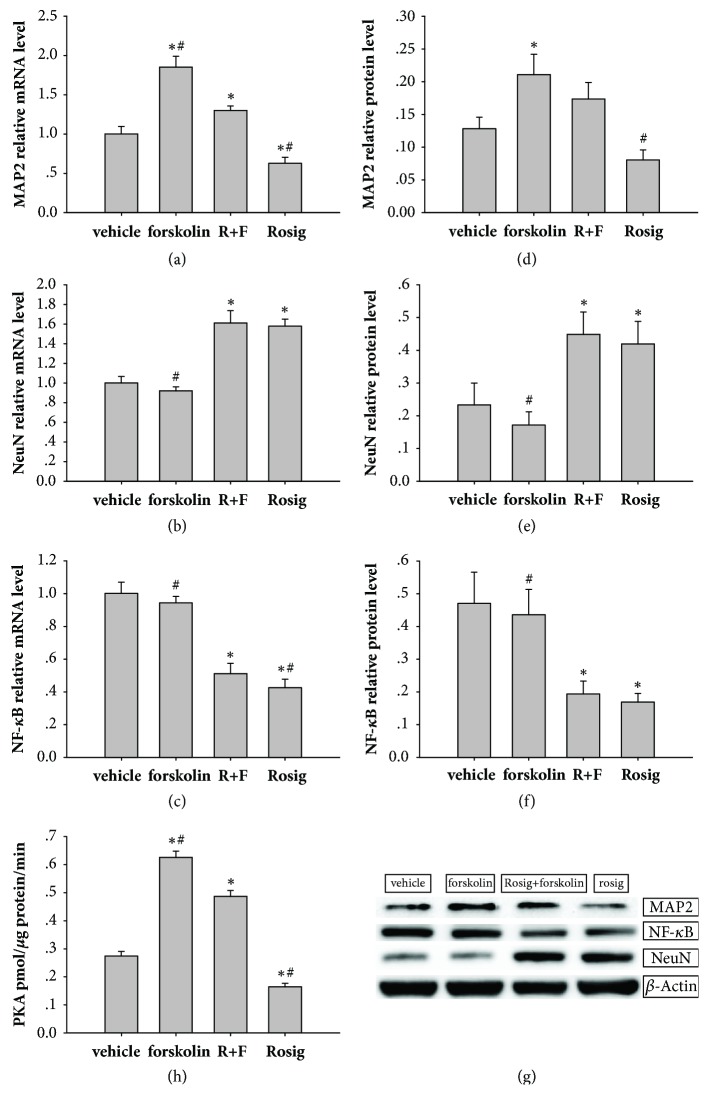
The mRNA and protein expression of MAP2, NeuN, and NF-*κ*B. The ELISA results of PKA. (a) Relative MAP2 mRNA expression levels. (b) Relative NeuN mRNA expression level. (c) Relative NF-*κ*B mRNA expression level. (d) Relative MAP2 protein expression level. (e) Relative NeuN protein expression level. (f) Relative NF-*κ*B protein expression level. (g) Representative western blots showing the effects of rosiglitazone, forskolin, and forskolin + rosiglitazone on MAP2, NF-*κ*B, and NeuN. (h) PKA expression levels detected by ELISA. Data were expressed as means±SD (n = 3). Each experiment was repeated at least three times. *∗* P < 0.05 compared with vehicle group. # P < 0.05, compared with the forskolin + rosiglitazone group.

On day 28 after spinal cord injury, when PKA were activated by forskolin the expression of MAP2 was higher in the forskolin group (1.85 ± 0.14) (Figure 5(a)) than in the other three groups (P < 0.01, n = 3). While PPAR-*γ* agonist rosiglitazone negatively regulates the neuronal formation, the rosiglitazone group (0.63 ± 0.08; P < 0.01, n = 3) showed the lowest expression of MAP2.

Rosiglitazone could inhibit the inflammation and promote the mature neuron survival, and when combined with PKA agonist forskolin, the expressions of mature neuron marker NeuN (Figure 5(b)) in the forskolin + rosiglitazone group (1.61±0.13) and the rosiglitazone group (1.58 ± 0.07) were higher than other groups. Using forskolin alone showed no protective effect on mature neuron survive; the lowest expression of NeuN was found in the forskolin group (0.92 ± 0.41).

Rosiglitazone is most critical in inhibiting the inflammatory response; the expression of inflammation marker NF-*κ*B (Figure 5(c)) was lowest in rosiglitazone group (0.43 ± 0.05). When PPAR-*γ* and PKA are simultaneously activated, the expression of NF-*κ*B in forskolin+ rosiglitazone group (0.51 ± 0.06) was still lower than forskolin group (0.94 ± 0.04) and the vehicle group (1.00 ± 0.07).

### 3.6. Relative Protein Expression of MAP2, NeuN, and NF-*κ*B following SCI

Indicators of each group in the WB experiment were the same as those in RT-PCR. MAP2 expression (Figures 5(d) and 5(j)) was higher in the forskolin group (0.21 ± 0.03) than in the vehicle (0.13 ± 0.02) and rosiglitazone (0.08 ± 0.02) groups. MAP2 expression was higher in the forskolin + rosiglitazone group (0.17 ± 0.03) than in the rosiglitazone group.

When the PPAR-*γ* was activated by rosiglitazone alone in rosiglitazone group (0.17 ± 0.03) and or PPAR-*γ* and PKA are simultaneously activated in forskolin + rosiglitazone groups (0.19 ± 0.04), the NF-*κ*B expression (Figures 5(f) and 5(j)) was lower than that in the other two groups (vehicle group: 0.47 ± 0.10, forskolin group: 0.44 ± 0.08). NF-*κ*B expression between the forskolin + rosiglitazone and rosiglitazone groups has no significant differences.

Rosiglitazone combined with forskolin has the best protective effect on mature neuron survival and the expression of NeuN (Figures 5(e) and 5(j)) in forskolin + rosiglitazone group (0.45 ± 0.07) compared with vehicle group (0.23 ± 0.07) and forskolin (0.17 ± 0.04) groups. When PPAR-*γ* was activated by rosiglitazone alone the expression of NeuN in rosiglitazone group (0.42 ± 0.07) was higher than forskolin and vehicle group.

### 3.7. Detection of PKA Expression by ELISA following SCI

PKA expression in forskolin group (0.63±0.02) was higher than other groups. This effect decreased when PPAR-*γ* and PKA are simultaneously activated (forskolin + rosiglitazone group: 0.49±0.02). But the forskolin + rosiglitazone group still showed higher PKA expression than that of the rosiglitazone (0.17±0.01) and vehicle groups (0.27±0.02) (Figure 5(h)). Considering the effect of rosiglitazone on NF-*κ*B, these data suggest that rosiglitazone likely inhibits PKA through NF-*κ*B.

### 3.8. Relative Protein Expression of PPAR-*γ* following SCI

**Figure 6:**
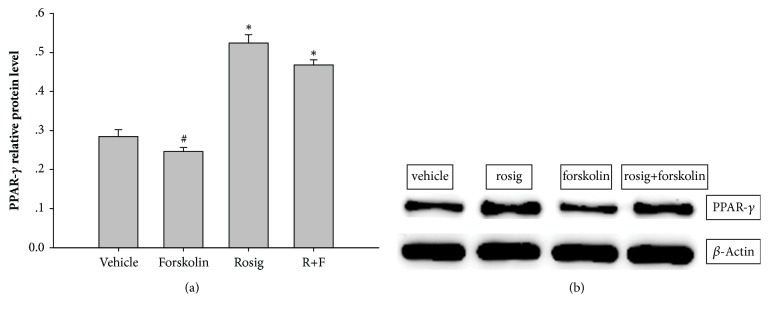
(a-b) The protein expression of PPAR-*γ* and representative western blots showing the effects of rosiglitazone, forskolin, and forskolin + rosiglitazone on PPAR-*γ*. Each experiment was repeated at least three times.

After rosiglitazone was used alone, the expression of PPAR-*γ* in rosiglitazone group (0.52±0.07) was higher than in vehicle (0.28±0.06) and forskolin groups (0.25±0.04). And no difference was found between rosiglitazone group and rosiglitazone + forskolin (R+F) group (0.47±0.04) (Figures 6(a)-6(b)).

## 4. Discussion

This study was undertaken to evaluate the effect of simultaneous activation of PPAR-*γ* and PKA on promoting locomotor recovery in SD rats, the expression of the inflammatory factor NF-*κ*B, survival of mature neurons, and the generation of newborn neurons after spinal cord injury. Our results indicate that activating PPAR-*γ* and PKA simultaneously could promote locomotor recovery and the neuronal formation after spinal cord injury. Compared to activating PPAR-*γ* or PKA individually, activating PPAR-*γ* and PKA simultaneously could also inhibit the expression of inflammatory factor NF-*κ*B and protect mature neurons.

There are few prolific sources of neural stem cells, including the telencephalon [29]. Lu et al. [30] reported the presence of neural stem cells around the ependymal membrane of the spinal cord. Our results verified the presence of neural stem cells in the spinal canal. Morales-Garcia [31] found that activation of PPAR-*γ* could promote neural stem cell proliferation in vitro; we could verify that the activation of PPAR-*γ* promotes proliferation of spinal cord-derived neural stem cells in vitro, which was reversed when PPAR-*γ* activation was inhibited.

The interaction of PPAR-*γ* and NF-*κ*B has been implicated in certain central neural diseases [32]. PPAR-*γ* can inhibit the activity of NF-*κ*B by binding to the subunits P65 and P50 of NF-*κ*B and competing for common transcription coactivators such as SRC-1 and p300/CBP (CREB-binding protein) or upregulate inhibitor kappa B (I*κ*B) protein, which prevents NF-*κ*B nuclear translocation which is a prerequisite for NF-*κ*B activation. PPAR-*γ* could also indirectly inhibit NF-*κ*B by activating transcription factor Nrf2, which reduces generation of prooxidative molecules that are required for NF-*κ*B activation. PPAR-*γ* therefore mediates the inhibition of NF-*κ*B, thereby reducing the central nervous system inflammatory cascade and effecting neuroprotection [33]. Under conditions of induced differentiation, we found that after PPAR-*γ* activation, the expression of NF-*κ*B is inhibited. PKA expression was also inhibited in this process and was restored when PPAR-*γ* was inhibited. This is likely related to PPAR*γ*-mediated inhibition of NF-*κ*B, since PKA is found downstream of NF-*κ*B; however, the feedback mechanism was not clear. LKB1, a substrate of PKA, plays an important role in axogenesis [34]. Koichiro et al. [35] suggested that the activation of PPAR-*γ* by rosiglitazone stimulated NSC growth and inhibited the differentiation of NSCs into neurons. We found that PPAR-*γ* activation in a neural stem cell differentiation induction system inhibits the MAP2 expression, which is consistent with the results of Koichiro. These results confirmed that the inhibitory effect of PPAR-*γ* on neuronal differentiation of spinal cord-derived neural stem cells could occur via NF-*κ*B and PKA in vitro. In order to show the inhibition of the cAMP pathway by rosiglitazone, cAMP levels in cells were measured by ELISA. And we see that changes in CAMP levels are consistent with changes in PKA levels.

In order to confirm PPAR-*γ* and PKA activation after SCI, the protein expression of PPAR-*γ* and PKA was observed in vivo. And we found a corresponding increase in the expression of PPAR-*γ* and PKA after applying rosiglitazone and forskolin, respectively. We used NeuN as an in vivo marker of mature neuronal survival following SCI. NeuN is an antigen used widely in research and diagnostics to identify postmitotic neurons. In the present study, we observed that the NeuN expression in the forskolin group was lower than that in the rosiglitazone and forskolin + rosiglitazone groups. This suggests that rosiglitazone inhibits inflammation and, combined with forskolin, promotes neuronal formation and has the best protective effect on mature neural stem cell survival. Forskolin alone had no effect on the survival of mature neurons in the inflammatory cascade following spinal cord injury.

MAP2 is a marker for dendritic lesions in CNS injury. MAP-2 is extremely sensitive to many factors, and recent investigations have revealed dynamic functions for MAP2 in the growth, differentiation, and plasticity of neurons. These discoveries indicate that modification and rearrangement of MAP2 are an early obligatory step in many processes that modify neuronal function [36]. In the present experiment study, we observed that the expression of MAP2 in the forskolin group was higher than that in the rosiglitazone and forskolin + rosiglitazone groups, which is likely related to the activation of PKA. PKA is implicated in the growth of axons and survival of newborn neurons [20, 21]. Thus, forskolin likely plays an important role in promoting neuron formation, while rosiglitazone negatively regulates neuron formation. We also examined the expression of cAMP* in vitro *and PKA* in vivo*, which was consistent with the expression of MAP2, suggesting that forskolin affects neuron formation through cAMP/PKA.

Compared with the rest of the control group, there was higher expression of NeuN in the spinal cord injury segment. Neuroprotection by genistein against oxidative stress injury in cortical neurons occurs via inhibition of NF-*κ*B, JNK, and ERK signaling pathway [37]. In the adult mammalian brain, NF-*κ*B activity seems to be crucial in regulating structural plasticity and replenishment of granule cells within the hippocampus throughout life. We suggest that inhibiting the expression of NF-*κ*B is a key strategy to inhibit the secondary inflammatory cascade and promote the locomotor recovery in SD rats after spinal cord injury. Further, the activation of PPAR-*γ* inhibits NF-*κ*B expression in a SD rat spinal cord injury model.

Genetic ablation of NF-*κ*B resulted in severe defects in the neurogenic region (dentate gyrus) of the hippocampus [19]. Reactivation of NF-*κ*B resulted in integration of newborn neurons and eventual regeneration of the dentate gyrus, accompanied by a complete recovery of structural and behavioral defects. These effects were attributed to NF-*κ*B-mediated control of PKA transcription, and regulation of axogenesis [19, 23]. Sulaiman et al. [38] found that forskolin could attenuate the adverse effects of long-term Schwann cell denervation on peripheral nerve regeneration* in vivo*. Further, forskolin had protective effects on behavioral deficits and neuropathological changes in a mouse model of cerebral amyloidosis [39] and could contribute to more efficient production of DAergic neurons from human-derived NPCs for therapy of neurodegenerative diseases [40]. In the present study, we found that forskolin could improve the number of nerve dendrites (MAP2 marker) in a SD rat spinal cord injury model, which was consistent with the aforementioned findings. During this process, we observed that forskolin increased the expression of PKA, which also confirmed that the effect of forskolin on neuronal formation is being through PKA.

In the present study, we found that simultaneous activation of PPAR-*γ* and PKA elicited the best results in terms of BBB score. However, simultaneous activation of PPAR-*γ* and PKA was not as efficacious as activating PKA alone in promoting the generation of newborn neurons and activating PPAR-*γ* alone in inhibiting inflammation. The recovery of locomotor function after spinal cord injury in rats is likely related to the secondary damage caused by the inflammatory cascade as well as neuronal cell regeneration after injury. The balance between the two is therefore important to achieve.

## 5. Conclusion

By combining the inhibition of NF-*κ*B and the survival and generation of neurons, activation of PPAR-*γ* and PKA simultaneously could produce the best results in terms of promoting motor function after spinal cord injury in SD rats. The underlying mechanism likely involves the PPAR-*γ*-mediated inhibition of NF-*κ*B and the consequent amelioration of the inflammatory cascade and PKA-mediated promotion of neuronal cell formation.

## Figures and Tables

**Table 1:** Quantity of NSCs (n = 3, mean ± SE).

	**Cell number (×10** ^**5**^ **)**	**Ratio (compared with vehicle group)**
Vehicle group	3.65 ± 0.13	1
Rosiglitazone group	4.58 ± 0.21*∗*	1.25
Rosiglitazone + G3335 group	3.57 ± 0.17	0.98

Comparison of the NSC number in the three groups. Each experiment was repeated at least three times. *∗* P < 0.05 (n = 3). There were significant differences between the rosiglitazone group and negative control group. Statistical methods: one-way ANOVA followed by pairwise comparison by the Holm-Sidak method.

## Data Availability

The data used to support the findings of this study are available from the corresponding author upon request.
